# Efficacy and safety of eribulin in patients with locally advanced or metastatic breast cancer not meeting trial eligibility criteria: a retrospective study

**DOI:** 10.1186/s12885-017-3846-8

**Published:** 2017-12-04

**Authors:** Sakura Iizumi, Tatsunori Shimoi, Natsuko Tsushita, Seiko Bun, Akihiko Shimomura, Emi Noguchi, Makoto Kodaira, Mayu Yunokawa, Kan Yonemori, Chikako Shimizu, Yasuhiro Fujiwara, Kenji Tamura

**Affiliations:** 1 Department of Breast and Medical Oncology, National Cancer Center Hospital, 5-1-1 Tsukiji, Chuo-ku, Tokyo, 104-0045 Japan; 2 Keio University Graduate School of Medicine, 160 Shinanomachi, Shinjuku-ku, Tokyo, 160-8582 Japan; 3 Course of Advanced Clinical Research of Cancer, Juntendo University Graduate School of Medicine, 3-1-3 Hongoh, bunkyo-ku, Tokyo, 113-0033 Japan; 4 Department of Pharmacy, National Cancer Center Hospital, 5-1-1 Tsukiji, Chuo-ku, Tokyo, 104-0045 Japan; 5 Department of Medical Oncology, Kodaira Hospital, 20-16 Sasameminamicho, Toda city, Saitama, 335-0035 Japan

**Keywords:** Breast cancer, Eribulin, Efficacy, Safety

## Abstract

**Background:**

The efficacy and safety of eribulin in patients with locally advanced or metastatic breast cancer has been demonstrated in phase III trials. However, as patients receiving eribulin in daily practice do not necessarily meet all the eligibility criteria of clinical trials, data for such patients are limited.

**Methods:**

We identified patients with locally advanced or metastatic breast cancer, treated with eribulin monotherapy between July 2011 and December 2015 at the National Cancer Center Hospital, Tokyo, Japan. Patients who would have met the following eligibility criteria from the EMBRACE trial were included in the eligible group, and the rest were included in the ineligible group: 1) Eastern Cooperative Oncology Group Performance status 0–2; 2) adequate function of principal organs; and 3) absence of active infection. We compared the relative dose intensity (RDI), tumor response, progression-free survival (PFS), overall survival (OS), and adverse events between the groups. Nominal and continuous values were compared using the Fisher’s exact test and Mann-Whitney U test, respectively. Survival outcomes were determined using Kaplan-Meier estimation, and between-group differences were assessed using the log-rank test.

**Results:**

Of the 203 patients included, 34 were classified into the ineligible group and 169 into the eligible group. Initial dose reduction and treatment discontinuation due to adverse events (AEs) were more common in the ineligible group (initial dose reduction: 23.5% in the ineligible group vs. 7.7% in the eligible group, *p* = 0.011; treatment discontinuation due to AEs: 11.8% vs. 3.0%, *p* = 0.045). However, RDI (66% vs. 71%, *p* = 0.130), response rate (15.6% vs. 18.1%, *p* = 1.000), PFS (3.7 months vs. 4.0 months, *p* = 0.913), OS (11.5 months vs. 16.1 months, *p* = 0.743), AEs requiring hospitalization (5.9% vs. 6.5%, p = 1.000), and grade 3/4 AEs were similar in both groups. PFS, OS, AEs requiring hospitalization, and discontinuation due to AEs in the eligible group were comparable to those found in previous phase III trials.

**Conclusion:**

The safety and efficacy of eribulin monotherapy was demonstrated in a broader patient population than that eligible for clinical trials. Eribulin may be a treatment option in these patients with locally advanced or metastatic breast cancer, considering dose reduction and pre-existing dysfunctions.

**Electronic supplementary material:**

The online version of this article (10.1186/s12885-017-3846-8) contains supplementary material, which is available to authorized users.

## Backgroud

Breast cancer is the most common cancer and leading cause of cancer mortality in women worldwide [1]. Despite recent developments in treatment, metastatic breast cancer remains incurable, and the 5-year survival rate is only 26% [2]. The goals of treatment are to prolong survival and to improve or maintain quality of life. Chemotherapy plays an important role, especially in patients with hormone-receptor negative or endocrine-resistant breast cancer. However, few chemotherapeutic agents have been shown to prolong overall survival (OS). While anthracyclines and taxanes are commonly used as standard first-line therapy in the metastatic setting, there is no single optimal subsequent-line chemotherapy [3].

Eribulin mesylate is a microtubule-inhibitor with a different mechanism from that of taxanes. It is the only chemotherapeutic agent shown to increase OS after treatment failure with anthracyclines and taxanes. Its efficacy and safety have been demonstrated in clinical trials, including two phase III trials, the EMBRACE and Study 301 [4, 5]. In addition to trial populations, its efficacy and safety in real-world populations have been reported in retrospective studies [6–10]. Also, a recently-published meta-analysis of retrospective series provides important clinical implications, by comparing the outcomes of eribulin in clinical practice and those from trials [11]. However, the efficacy and safety, specifically in patients who would not have participated in clinical trials, but who still receive eribulin in daily practice, have not been reported.

We conducted a retrospective study to assess the efficacy and safety of eribulin in patients with locally advanced or metastatic breast cancer who would not have met the eligibility criteria of the EMBRACE trial.

## Methods

### Patients

We retrieved the medical records of patients with locally advanced or metastatic, pathologically confirmed breast cancer treated with eribulin monotherapy between July 2011 and December 2015 at the National Cancer Center Hospital (Tokyo, Japan). Patients who received eribulin at different hospitals before being treated at our hospital or those with insufficient data regarding trial eligibility were excluded from the analysis.

### Definition of patient analysis groups (trial eligible and trial ineligible)

We determined the eligibility of patients included in this analysis according to the following criteria based on the EMBRACE trial: 1) Eastern Cooperative Oncology Group Performance status (PS) of 0 to 2; 2) adequate function of principal organs (adequate bone marrow, renal, and liver function as evidenced by absolute neutrophil count ≥1500/mm^3^, platelets ≥10 × 10^4^/mm^3^, hemoglobin ≥10 g/dL, serum creatinine ≤2.0 mg/dL, total bilirubin ≤1.5 times the upper limit of normal [ULN], and aspartate transaminase [AST] and alanine transaminase [ALT] ≤ three times ULN, [AST and ALT ≤ five times ULN in patients with liver metastasis]); and 3) absence of active infection. Patients satisfying all these criteria were classified into the “eligible group”; patients not satisfying any one of these criteria were classified into the “ineligible group.” We selected the criteria that may independently affect the efficacy (or dose intensity [DI]) or safety of eribulin and that were well-documented by the medical records. We considered that factors regarding prior chemotherapy (including the no. of prior chemotherapy lines) do not affect the efficacy or safety of eribulin by themselves; thus, we did not include them for the criteria for the classification. Life expectancy, stable brain metastasis, and peripheral neuropathy were not included because they were not necessarily well-documented at the initiation of eribulin.

### Treatment

Patients received intravenous infusions of eribulin mesylate 1.4 mg/m^2^ over 2–5 min on days 1 and 8 of each 21-day cycle. The dosing was adjusted according to dose modification recommended by the FDA prescribing information [12], adverse events, or the physicians’ judgment. Treatment cycles were repeated until progressive disease or unacceptable toxicity, or until the patient decided to terminate treatment.

### Assessment

We assessed tumor response according to the Response Evaluation Criteria in Solid Tumors (RECIST), version 1.1 [13] by using computed tomography (CT) scans. CT scans were obtained every other cycle or sooner if needed. Confirmation of response was not required. Response rate (RR) and disease control rate (DCR) were defined as proportions of patients who achieved at least partial response and stable disease as best response, respectively. Progression-free survival (PFS) was defined as the time from the initiation of eribulin monotherapy until either clinical or objective disease progression, or death. OS was defined as the time from the initiation of eribulin monotherapy until death. We also assessed relative dose intensity (RDI), DI and planned dose intensity (PDI) according to the following formula:

RDI (%) = DI/PDI ×100,

DI (mg/week) = Cumulative dose/treatment duration,

PDI (mg/week) =1.4 × 2/3

Adverse events (AEs) were assessed using the Common Terminology Criteria for Adverse Events (CTCAE), version 4.0.

### Statistical analysis

The study was designed to compare the efficacy (RR, DCR, PFS, and OS) and safety (frequency of grade 3 or worse AEs, discontinuation due to AEs, and AEs requiring hospitalization) of eribulin between the eligible group and the ineligible group. Nominal values and continuous values were compared using Fisher’s exact test and Mann-Whitney U test, respectively. Only patients with target lesions were analyzed for RR and DCR. Survival outcomes were obtained using Kaplan-Meier estimates, and the differences between the two groups were assessed using log-rank test. Tests were considered significant if the two-sided *p*-value was <0.05. Analyses were performed with EZR (Saitama Medical Center, Jichi Medical University, Saitama, Japan), which is a graphical user interface for R (The R foundation for Statistical Computing, Vienna, Austria) [14].

## Results

### Patients

**Fig. 1:**
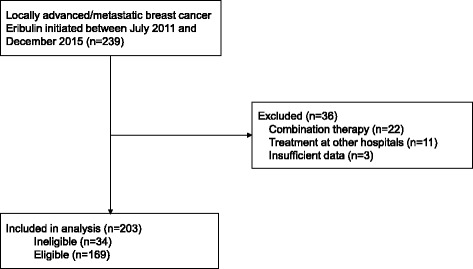
Flow diagram of the patient selection process

A total of 203 patients were included in the analysis: 34 were included in the ineligible group and 169 in the eligible group (Fig. 1). Baseline patient characteristics are shown in Table 1. The proportion of patients with hormone receptor-positive breast cancer (estrogen receptor + and/or progesterone receptor+) was lower in the eligible group than in the ineligible group (58.8% vs. 79.3%, *p* = 0.024); otherwise, there was no significant difference in patient characteristics between groups.

**Table 1:** Patient characteristics by group

Characteristic		Ineligible (*n* = 34)	Eligible (*n* = 169)	Total (*n* = 203)	*P*-value*
Median age	Median [range]	54.0 [31–77]	58.0 [30–81]	58.0 [30–81]	0.179
Performance status (%)	0	18 (52.9)	88 (52.1)	106 (52.2)	0.231
	1	13 (38.2)	74 (43.8)	87 (42.9)	
	2	2 (5.9)	7 (4.1)	9 (4.4)	
	3	1 (2.9)	0 (0.0)	1 (0.5)	
Hormone receptor positive (%)		20 (58.8)	134 (79.3)	154 (75.9)	0.024
Her2+ (%)^a^		4 (11.8)	23 (13.6)	27 (13.3)	1.000
Metastatic sites ≥3 (%)		13 (38.2)	56 (33.1)	69 (34.0)	0.559
Liver metastasis (%)		24 (70.6)	93 (55.0)	117 (57.6)	0.128
Lung metastasis (%)		17 (50.0)	69 (40.8)	86 (42.4)	0.346
Bone only (%)		1 (2.9)	2 (1.2)	3 (1.5)	0.425
Target lesion present (%)		32 (94.1)	144 (85.2)	26 (12.8)	0.387
Prior surgery (%)		30 (88.2)	139 (82.2)	169 (83.3)	0.462
No. of prior chemotherapy lines	Median [range]	3 [2–8]	3 [1–11]^b^	3 [1–11]	0.294
No. of prior chemotherapy lines for advanced disease	Median [range]	2 [0–7]	2 [0–9]	2 [0–9]	0.204

Nominal values and continuous values were compared using Fisher’s exact test and Mann-Whitney U test, respectively

*Ineligible vs. eligible

^a^Two patients in the eligible group had decreased ejection fraction. The rest of the patients had received multiple lines of Her2-targeted regimens

^b^Three patients with cardiac dysfunction had received only one prior chemotherapy regimen (a taxane)

### Worst CTCAE grade leading to exclusion from the eligible group

Table 2 shows the numbers of patients who did not meet each of the eligibility criteria, with the worst grade of the pre-existing dysfunction for each criterion (shown by CTCAE, version 4.0 where appropriate). The worst grade for pre-existing dysfunctions in the ineligible group was grade 2 for all unmet criteria, except for increased AST/ALT levels (grade 3). All patients had PS of 0–2, except for one patient (2.9%) with a PS of 3 because of pain from bone metastases. One patient had an infection of a cutaneous metastasis, which required antibiotic administration.

**Table 2:** Unmet eligibility criteria with the worst grade in the ineligible group

Eligibility criteria	N (%)	Worst grade
Hemoglobin <10 g/dL	12 (35.3)	G2^a^
Absolute neutrophil count <1500/mm^3^	11 (32.4)	G2^b^
Platelets <10 × 10^4^/mm^3^	4 (11.8)	G2^c^
Total bilirubin >1.5 x ULN	1 (2.9)	G2
AST or ALT >3 x ULN (5 x ULN in liver metastasis)	5 (14.7)	G3
Creatinine >2.0 mg/dL	0 (0.0)	–
Active infection	1 (2.9)	G2^d^

Abbreviations: ULN, upper limit of normal; AST, aspartate aminotransferase; ALT, alanine aminotransferase; G, grade. No patient received interventions for decreased hemoglobin, absolute neutrophil count, or platelets

^a^Mean, 9.3 g/dL; range, 8.2–9.9 g/dL. The etiology of anemia was chemotherapy in 10 patients and cancer in 2 patients

^b^Mean, 1400/mm^3^; range, 1080–1470/mm^3^. Initial dose of eribulin was reduced in one patient

^c^Mean, 7.8 × 10^4^/mm^3^; range, 7.1–9.6 × 10^4^/mm^3^

^d^Infection of cutaneous metastasis requiring an oral antibiotic. This patient also had hemoglobin <10 g/dL

### Relative dose intensity, cumulative dose, and initial dose reduction

The initial dose was reduced more frequently in the ineligible group than in the eligible group (8/34 [23.5%] vs. 13/169 [7.7%], *p* = 0.011). However, there was no significant difference in RDI (%) between the ineligible group and the eligible group (median: 66, range: 38–97 vs. median 72, range 27–102; *p* = 0.130) or cumulative dose (mg) (median: 14.3, range: 2.0–59.5 vs. median: 16.0, range: 1.5–109.2; *p* = 0.389) (Additional file 1: Table S1).

### Efficacy

There was no significant difference in RR between the ineligible group and the eligible group (15.6%; 95% confidence interval [CI], 5.3–32.8% vs. 18.1%; 95% CI, 12.1–25.3%; *p* = 1.000) or DCR (65.6%; 95% CI, 46.8–81.4% vs. 62.5%; 95% CI, 54.1–70.4%; *p* = 0.841) (Additional file 2: Table S2). The RR and DCR in the total population were 17.6% (95% CI, 12.3–24.1%) and 63.1% (95% CI, 55.5–70.2%), respectively.

**Fig. 2:**
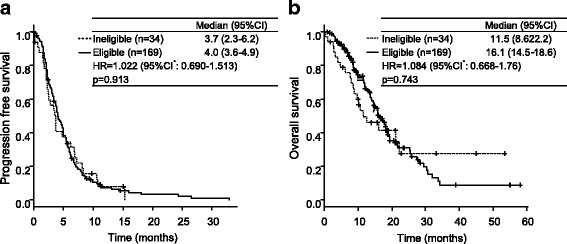
Kaplan-Meier curves showing (**a**) progression-free survival and (**b**) overall survival. CI: confidence interval; HR: hazard ratio

Figure 2 shows the Kaplan-Meier curves for PFS and OS. There was no significant difference between the ineligible group and the eligible group in PFS (median: 3.7 months vs. 4.0 months, *p* = 0.913) or in OS (median: 11.5 months vs. 16.1 months, *p* = 0.743). The medians for PFS and OS in the total population were 4.0 months (95% CI, 3.5–4.8 months) and 15.9 months (95% CI, 13.8–18.5 months), respectively. The proportion of patients who received treatment after eribulin was 52.9% in the ineligible group and 65.1% in the eligible group.

### Safety

Table 3 shows the frequency of AEs by group. There was no significant difference between the two groups in any of the AE parameters, except for discontinuation of treatment due to AEs (ineligible group: 4 [11.8%] vs. eligible group: 5 [3.0%], *p* = 0.045). The AEs leading to treatment discontinuation were as follows (ineligible group vs. eligible group, respectively): febrile neutropenia in 4 vs. 0 (2.9% vs. 0.0%), peripheral neuropathy in 1 vs. 3 (2.9% vs. 1.8%), increased aminotransferases in 1 vs. 0 (2.9% vs. 0.0%), anorexia in 0 vs. 2 (0.0% vs. 1.2%), and neutropenia in 1 vs. 0 (2.9% vs. 0.0%). The unmet criteria in ineligible patients who discontinued treatment due to AEs were ECOG PS, absolute neutrophil count, hemoglobin, and total bilirubin (one patient for each criterion). Adverse events by eligibility criteria in the ineligible group are shown in Additional file 3: Table S3).

**Table 3:** Adverse events by group

Grade 3 or 4 AEs	Ineligible (*n* = 34)	Eligible (*n* = 169)	Total (*n* = 203)	*P*-value*
Any (%)	25 (73.5)	113 (66.9)	138 (68.0)	0.547
Hematological				
Leukopenia (%)	13 (38.2)	67 (39.6)	80 (39.4)	1.000
Neutropenia (%)	21 (61.8)	103 (60.9)	124 (61.1)	1.000
Thrombocytopenia (%)	0 (0.0)	0 (0.0)	0 (0.0)	NA
Anemia (%)	4 (11.8)	6 (3.6)	10 (4.9)	0.066
Febrile neutropenia (%)	4 (11.8)	14 (8.3)	18 (8.9)	0.512
Non-hematological				
Fatigue	0 (0.0)	0 (0.0)	0 (0.0)	NA
Peripheral neuropathy	0 (0.0)	0 (0.0)	0 (0.0)	NA
Nausea	0 (0.0)	0 (0.0)	0 (0.0)	NA
Constipation	0 (0.0)	0 (0.0)	0 (0.0)	NA
Diarrhea	0 (0.0)	0 (0.0)	0 (0.0)	NA
Total Bilirubin (%)	1 (2.9)	0 (0.0)	1 (0.5)	0.167
AST increased (%)	1 (2.9)	5 (3.0)	6 (3.0)	1.000
ALT increased (%)	2 (5.9)	4 (2.4)	6 (3.0)	0.264
Discontinuation due to AEs (%)	4 (11.8)	5 (3.0)	9 (4.4)	0.045
AEs leading to hospitalization (%)	2 (5.9)	11 (6.5)	13 (6.4)	1.000

Abbreviations: AE, adverse event; AST, aspartate aminotransferase; ALT, alanine aminotransferase; NA, not assessed

^*^Ineligible vs. eligible

## Discussion

In this study, we retrospectively evaluated the efficacy and safety of eribulin monotherapy for the treatment of locally advanced or metastatic breast cancer in patients who would not have been eligible to participate in phase III clinical trials. Tumor response, PFS, and OS did not differ significantly between the ineligible and eligible groups. Although initial dose reduction and treatment discontinuation due to AEs were more common in the ineligible group than in the eligible group, RDI and severe AEs did not differ significantly between the two groups.

The results in the eligible group were comparable to those of clinical trials (PFS: 3.7–4.1 months, OS: 13.1–15.9 months, AEs requiring hospitalization: 13.4%, discontinuation due to AEs: 7.9–13%) [4, 5]. This confirms the external validity of this study and helps interpret the outcomes in the ineligible group.

RR, PFS, and OS did not differ significantly between the ineligible and eligible groups. As tumor response and survival outcomes were similar between the two groups in the current study, eribulin may benefit patients with poorer baseline dysfunctions.

There was no significant difference in RDI between the two groups, although an initial dose reduction was more frequent in the ineligible group. This indicates that the eligible patients experienced dose reduction or change in dosing schedule at some point during treatment, even if they did not experience dose reduction for the first administration. Although higher RDI might contribute to better survival, as reported for first-line chemotherapy with anthracyclines or taxanes [15], initial dose reduction might be acceptable considering the limited impact on RDI.

There were no significant differences between the safety profiles of the groups, apart from discontinuation due to AEs, which was more common in the ineligible group. Despite this finding, the observed frequency of discontinuation in the ineligible group was within the range observed in the phase III trials [4, 5]. There seemed to be differences in individual AE items between the eligible patients in this study and patients in clinical trials, possibly due to differences in the frequency of assessments and the retrospective nature of this study. However, for both study groups, clinically significant safety outcomes such as treatment discontinuation due to AEs or AEs requiring hospitalization, seemed equivalent to or lower than those reported in the clinical trials [4, 5]. These results suggest that eribulin can be used safely in patients who would be considered ineligible for clinical trials, although AEs may have to be monitored with greater caution.

Despite these positive findings, this study has limitations. In patients with a poorer baseline condition at treatment initiation compared with those in the ineligible group, the benefit and safety of eribulin remain unclear. Most of the ineligible patients in this study had a preserved PS, and their pre-existing dysfunctions were no worse than grade 2 (by CTCAE v4.0), with the exception of increased aminotransferases, and therefore, outcomes in patients with grade 3 or worse conditions remain unknown. Furthermore, only conserved PS and organ functions and absence of active infection were used to classify patients into the ineligible and eligible groups in this study. Patients who would have been deemed ineligible on the basis of the other criteria used in the EMBRACE trial were not included in the ineligible group in our study. Differences might also exist among physicians in the selection of patients for eribulin treatment, as uniform assessment criteria were not used to judge the suitability of eribulin. Despite these limitations, the current study provides data in a real world setting and will help future clinical practice.

## Conclusions

The safety and efficacy of eribulin monotherapy has been demonstrated in patients who would have been considered ineligible for the clinical trials. Eribulin presents a viable treatment option for locally advanced or metastatic breast cancer patients with preserved PS, when used with consideration of dose reduction and caution regarding the degree of pre-existing dysfunctions and AEs. Efficacy and safety for each eligibility criterion should be assessed in a larger number of patients.

## Additional files

Additional file 1: Table S1. Relative dose intensity, cumulative dose, and initial dose reduction. (DOCX 18 kb)

Additional file 2: Table S2. Tumor response. (DOCX 19 kb)

Additional file 3: Table S2. Grade 3 or 4 adverse events by unmet factor of eligibility criteria in the ineligible group (*n* = 34). (DOCX 22 kb)

## Abbreviations

AE: adverse event
ALT: alanine aminotransferase
AST: aspartate aminotransferase
CI: confidence interval
CR: complete response
CTCAE: Common Terminology Criteria for Adverse Events
DCR: disease control rate
DI: dose intensity
ECOG: Eastern Cooperative Oncology Group
G: grade
NA: not assessed
NE: not evaluable
OS: overall survival
PD: progressive disease
PDI: planned dose intensity
PFS: progression-free survival
PR: partial response
PS: performance status
RDI: relative dose intensity
RECIST: Response Evaluation Criteria in Solid Tumors
RR: response rate; SD, stable disease
