# Supplementary material for: Efficacy and safety of eribulin in patients with locally advanced or metastatic breast cancer not meeting trial eligibility criteria: a retrospective study
Source: BMC Cancer. 2017 Dec 4;17:819. doi: 10.1186/s12885-017-3846-8 (PMC5716387; doi:10.1186/s12885-017-3846-8)
Supplement: Supplementary file 1 — Relative dose intensity, cumulative dose, and initial dose reduction. (DOCX 18 kb) [file 12885_2017_3846_MOESM1_ESM.docx]

Additional file 1: Table S1. Relative dose intensity, cumulative dose, and initial dose reduction.

|  |  | Ineligible group  (n=34) | Eligible group  (n=169) | P-value |
| --- | --- | --- | --- | --- |
| RDI | Median [range] | 66 [38-97] | 71 [27-102] | 0.130 |
| Cumulative dose (mg) | Median [range] | 14.3 [2.0-59.5] | 16.0 [1.5-109.2] | 0.389 |
| Initial dose reduction | (%) | 8 (23.5) | 13 ( 7.7) | 0.011 |
|  |  |  |  |  |
| Initial dose (mg/m^2^) | 1.4(%) | 26 (76.5) | 156 (92.3) |  |
|  | 1.1(%) | 6 (17.6) | 10 ( 5.9) |  |
|  | 0.9(%) | 0 ( 0.0) | 1 ( 0.6) |  |
|  | 0.7(%) | 2 ( 5.9) | 2 ( 1.2) |  |
