# Supplementary material for: Efficacy and safety of eribulin in patients with locally advanced or metastatic breast cancer not meeting trial eligibility criteria: a retrospective study
Source: BMC Cancer. 2017 Dec 4;17:819. doi: 10.1186/s12885-017-3846-8 (PMC5716387; doi:10.1186/s12885-017-3846-8)
Supplement: Supplementary file 2 — Tumor response. (DOCX 19 kb) [file 12885_2017_3846_MOESM2_ESM.docx]

Additional file 2: Table S2. Tumor response.

| Best overall response | Ineligible group  (n=32) | Eligible group (n=144) | P-value |
| --- | --- | --- | --- |
| CR (%) | 1 ( 3.1) | 1 ( 0.7) |  |
| PR (%) | 4 (12.5) | 25 (17.4) |  |
| SD (%) | 16 (50.0) | 64 (44.4) |  |
| PD (%) | 10 (31.2) | 50 (34.7) |  |
| NE (%) | 1 ( 3.1) | 4 ( 2.8) |  |
| RR (%) | 5 (15.6) | 26 (18.1) | 1.000 |
| DCR (%) | 21 (65.6) | 90 (62.5) | 0.841 |

Abbreviations: CR, complete response; PR, partial response; SD, stable disease; PD, progressive disease; NE, not evaluable; RR, response rate; DCR, disease control rate.
