# Supplementary material for: Efficacy and safety of eribulin in patients with locally advanced or metastatic breast cancer not meeting trial eligibility criteria: a retrospective study
Source: BMC Cancer. 2017 Dec 4;17:819. doi: 10.1186/s12885-017-3846-8 (PMC5716387; doi:10.1186/s12885-017-3846-8)
Supplement: Supplementary file 3 — Grade 3 or 4 adverse events by unmet factor of eligibility criteria in the ineligible group (n = 34). (DOCX 22 kb) [file 12885_2017_3846_MOESM3_ESM.docx]

Additional file 3: Table S3. Grade 3 or 4 adverse events by unmet factor of eligibility criteria in the ineligible group (n=34)

|  | PS  n=1 | Hb  n=12 | ANC  n=11 | Platelets  n=4 | T. Bil  n=1 | AST/ALT  n=5 | Infection*  n=1 |
| --- | --- | --- | --- | --- | --- | --- | --- |
| Any adverse event (%) | 1 (100.0) | 8 ( 66.7) | 10 ( 90.9) | 2 ( 50.0) | 1 (100.0) | 3 ( 60.0) | 0 (0.0) |
| Hematological |  |  |  |  |  |  |  |
| Leukopenia (%) | 1 (100.0) | 5 ( 41.7) | 6 ( 54.5) | 0 (0.0) | 0 (0.0) | 1 ( 20.0) | 0 (0.0) |
| Neutropenia (%) | 1 (100.0) | 7 ( 58.3) | 0 (0.0) | 1 ( 25.0) | 1 (100.0) | 1 ( 20.0) | 0 (0.0) |
| Thrombocytopenia (%) | 0 (0.0) | 0 ( 0.0) | 0 (0.0) | 0 (0.0) | 0 (0.0) | 0 (0.0) | 0 (0.0) |
| Anemia (%) | 0 (0.0) | 3 ( 25.0) | 0 (0.0) | 1 ( 25.0) | 0 (0.0) | 0 (0.0) | 0 (0.0) |
| Febrile neutropenia (%) | 1 (100.0) | 3 ( 25.0) | 0 (0.0) | 0 (0.0) | 0 (0.0) | 0 (0.0) | 0 (0.0) |
| Non-hematological |  |  |  |  |  |  |  |
| Fatigue | 0 (0.0) | 0 (0.0) | 0 (0.0) | 0 (0.0) | 0 (0.0) | 0 (0.0) | 0 (0.0) |
| Peripheral neuropathy | 0 (0.0) | 0 (0.0) | 0 (0.0) | 0 (0.0) | 0 (0.0) | 0 (0.0) | 0 (0.0) |
| Nausea | 0 (0.0) | 0 (0.0) | 0 (0.0) | 0 (0.0) | 0 (0.0) | 0 (0.0) | 0 (0.0) |
| Constipation | 0 (0.0) | 0 (0.0) | 0 (0.0) | 0 (0.0) | 0 (0.0) | 0 (0.0) | 0 (0.0) |
| Diarrhea | 0 (0.0) | 0 (0.0) | 0 (0.0) | 0 (0.0) | 0 (0.0) | 0 (0.0) | 0 (0.0) |
| Total Bilirubin (%) | 0 (0.0) | 0 (0.0) | 0 (0.0) | 0 (0.0) | 0 (0.0) | 1 ( 20.0) | 0 (0.0) |
| AST increased (%) | 0 (0.0) | 0 (0.0) | 0 (0.0) | 0 (0.0) | 0 (0.0) | 1 ( 20.0) | 0 (0.0) |
| ALT increased (%) | 0 (0.0) | 0 (0.0) | 0 (0.0) | 0 (0.0) | 0 (0.0) | 2 ( 40.0) | 0 (0.0) |
| Discontinuation† (%) | 1 (100.0) | 1 (8.3) | 1 (9.1) | 0 (0.0) | 1 (100.0) | 0 (0.0) | 0 (0.0) |
| Hospitalization‡ (%) | 1 (100.0) | 1 (8.3) | 0 (0.0) | 0 (0.0) | 0 (0.0) | 0 (0.0) | 0 (0.0) |

*This patient also had hemoglobin < 10 g/dL. †Discontinuation due to adverse events. ‡adverse events leading to hospitalization. Abbreviations: PS, performance status, Hb, hemoglobin, ANC, absolute neutrophil count; T. Bil, total bilirubin; AST, aspartate aminotransferase; ALT, alanine aminotransferase.
